# Evaluation of Melatonin Therapy in Patients with Myocardial Ischemia-Reperfusion Injury: A Systematic Review and Meta-Analysis

**DOI:** 10.1155/2022/4610522

**Published:** 2022-03-03

**Authors:** Tingting Lv, Junwei Yan, Yunwei Lou, Zeying Zhang, Mengfei Ye, Jiedong Zhou, Fangyi Luo, Chenchen Bi, Hui Lin, Jian Zhang, Hangyuan Guo, Zheng Liu

**Affiliations:** ^1^Department of Pharmacology, Medical School of Shaoxing University, Shaoxing, Zhejiang, China; ^2^Department of Cardiology, Shaoxing People's Hospital (Shaoxing Hospital, Zhejiang University School of Medicine), Shaoxing, Zhejiang, China; ^3^Department of Psychiatry, Shaoxing Seventh People's Hospital, Shaoxing, Zhejiang, China; ^4^Department of Clinical Medicine, Medical School of Shaoxing University, Shaoxing, Zhejiang, China

## Abstract

**Objectives:**

We conducted a meta-analysis to quantitatively evaluate the effect of melatonin therapy on patients with myocardial ischemia-reperfusion injury (MIRI) and explore the influencing factors.

**Background:**

Although preclinical studies have shown that melatonin can alleviate MIRI, its protective effect on MIRI in patients remains controversial.

**Methods:**

We searched PubMed, the Cochrane Library, and Embase. The primary outcome was cardiac function (left ventricular ejection fraction [LVEF], left ventricular end-diastolic volume [LVEDV], and left ventricular end-systolic volume [LVESV]) and myocardial infarct parameters (total left ventricular mass and infarct size).

**Results:**

We included nine randomized controlled clinical trials with 631 subjects. Our results showed that melatonin had no significant effects on the primary outcome, but subgroup analyses indicated that when melatonin was administered by intravenous and intracoronary injection at the early stage of myocardial ischemia, LVEF was improved (<3.5 h; standardized mean difference [SMD]:0.50; 95% CI: 0.06 to 0.94; *P* = 0.03) and the infarct size was reduced (<2.5 h, SMD: −0.86; 95% CI: −1.51 to −0.22; *P* = 0.01), whereas when melatonin was injected at the late stage of myocardial ischemia (≥3.5 h or 2.5 h), the results were the opposite. Furthermore, melatonin intervention reduced the level of cardiac injury markers, inflammatory cytokines, oxidation factors, and increased the level of antioxidant factors (*P* < 0.001).

**Conclusions:**

The results indicated that the cardioprotective function of melatonin for MIRI was influenced by the route and timing regimen of melatonin administration; the mechanism of which may be associated with the production of inflammatory cytokines, the balance of oxidation, and antioxidant factors.

## 1. Introduction

Acute myocardial infarction (AMI) is a leading cause of disability and mortality worldwide and is responsible for a third of all deaths in developed countries annually [1]. Rapid restoration of coronary blood flow is the key to salvage the endangered myocardium [2, 3]. Currently, the most effective and prompt methods of revascularization are thrombolysis, primary percutaneous coronary intervention (pPCI), and coronary artery bypass graft (CABG), all of which have been proven to reduce infarct size and mortality. However, subsequent myocardial reperfusion itself can lead to further myocardial damage, which accounts for up to 50% of the final myocardial injury [4–9]. Fast restoration of myocardial blood flow may induce myocardial injury (impaired myocardial contractility), ventricular arrhythmias, and microvascular dysfunction; this phenomenon is called myocardial ischemia-reperfusion injury (MIRI), which is strongly associated with adverse prognosis [5, 10–12]. Therefore, therapeutic strategies for preventing and alleviating MIRI have become crucial in clinical practice [13, 14]. However, there is still a lack of effective pharmacological treatments targeting MIRI [15].

Melatonin (N-acetyl-5-methoxytryptamine), a circadian hormone secreted mainly by the pineal gland, was first recognized for its ability to regulate circadian rhythms [16, 17]. Emerging evidence has shown that melatonin is a pleiotropic hormone with many functions, including hypnotic, sedative, anxiolytic, antioxidation, anti-inflammatory, and cardioprotective effects [18–20]. Among them, cardioprotective function against various pathological stimuli of melatonin has attracted great attention [21]. Preclinical studies and two meta-analyses have shown the ability of melatonin to alleviate MIRI and explored the potential mechanism, which mainly involved free radical scavenging and upregulation of antioxidant systems [22–26]. However, several studies have shown inconsistent results and the underlying influencing factors are unclear [27, 28], with some even suggesting that melatonin may aggravate MIRI under certain conditions [29]. Therefore, the role of melatonin and its potential mechanism and influencing factors in patients with MIRI remains to be further evaluated.

In this study, we aimed to quantitatively evaluate the effect of melatonin therapy on patients with MIRI and explore the underlying molecular and cellular mechanisms from the available evidence of the included clinical studies. In addition, we sought to identify the most appropriate route and timing regimen of melatonin administration.

## 2. Methods

### 2.1. Literature Search Strategy

We conducted a systematic literature search of three electronic databases (PubMed, the Cochrane Library, and Embase) from inception until June 2021 to identify potentially relevant studies on the influence of melatonin therapy on patients with MIRI. The search terms used Medical Subject Headings (MeSH) and free-text terms for “melatonin” and “coronary artery disease” (Box 1). We searched the references of the included studies, as well as the comments and conference abstracts to ensure that our search strategy discovered all pertinent articles. The study language was restricted to English. Two authors (JWY and YWL) removed duplicates and screened the records to ensure that all records were independently evaluated. Any differences in opinions were resolved through discussion with a third author (TTL).

### 2.2. Inclusion and Exclusion Criteria

All of the articles eligible for inclusion strictly met the criteria of the Population, Intervention, Comparison, Outcome, and Study design (PICOS) framework according to the Preferred Reporting Items for Systematic Reviews and Meta-Analyses (PRISMA) recommendations [30]. The inclusion criteria were as follows: (1) participants: adult patients with ischemic heart diseases (IHD); (2) intervention and comparison: melatonin and its analogs, including agomelatine, tasimelteon, and ramelteon (rozerem) compared to vehicle treatment; (3) outcome: primary outcome reporting left ventricular (LV) function and secondary outcome reporting cardiovascular blood markers; and (4) study design: randomized controlled trials (RCTs). Studies without full text or primary data that could not be obtained were excluded.

### 2.3. Data Extraction

Two investigators (JWY and TTL) independently read the included articles to extract the experimental details and data. The following information was acquired: the first author, publication year, country, population characteristics (i.e., sample size, sex, age, and diagnosis of patients), specific therapeutic strategy (i.e., timing, dosage, and route of melatonin administration), mean duration of ischemia reperfusion injury, follow-up duration, and detailed primary and secondary outcome measures. We extracted data including the means, standard deviation (SD) of the means, and the sample size in each group. When the exact data were unavailable, only graphs and bar charts, we turned to the author for unpublished data. If this was unsuccessful, a digital ruler was used to estimate the data from the graphs or bar charts. The original data were estimated based on the coordinate axis [31, 32]. Any disagreement was resolved by discussion with a third author until a final consensus was reached.

### 2.4. Risk-of-Bias and Quality Assessment

The included articles were appraised and graded independently by two authors (JWY and YWL) based on the Cochrane collaboration tool [33]. The following aspects were judged as “low,” “high,” or “unclear” risk: selection bias, performance bias, detection bias, attrition bias, reporting bias, and other biases. Discrepancies were resolved by discussion with a third author (TTL) until a final consensus was reached.

### 2.5. Data Synthesis and Analysis

We measured the impact of melatonin intervention by the standardized mean difference (SMD) of the post-intervention variables of cardiac function and cardiovascular blood markers. The SMD was assessed using Cohen's as a measure of effect size; 0.2–0.5 was viewed as small, 0.5–0.8 as medium, and ≥0.8 as large. The continuous variable outcomes were described as effect size ± 95% confidence intervals (CIs). We used a random-effects model because we assumed that the effect sizes of the included articles were similar but not identical because of the predictable heterogeneity between studies (i.e., different outcome measures and melatonin therapeutic strategies) [34].

The heterogeneity across the studies was analyzed by Cochran's *Q* test and *I*^2^ statistics. *I*^2^ values of 25%–50%, 50%–75%, and ≥75% denoted low, moderate, and high heterogeneity, respectively [35]. Subgroup analyses were performed to evaluate whether the effect size was influenced by the mode of administration and to access heterogeneity. Sensitivity analyses were also conducted to assess whether the results were influenced substantially by a single study.

Cumulative meta-analyses were performed to evaluate the effect of the mean time from symptoms to melatonin intracoronary injection and follow-up duration on the pooled estimates. In short, in the cumulative meta-analysis, based on the mean time from symptoms to melatonin intracoronary injection (from early to late), studies were added individually and the results showed the pooled estimate each time the results of a new study were added. Statistical analyses were performed using Stata software version 12.0 (Stata Corp, College Station, TX, USA) and *P*-values <0.05 were considered to be statistically significant.

## 3. Results

### 3.1. Study Characteristics

**Figure 1:**
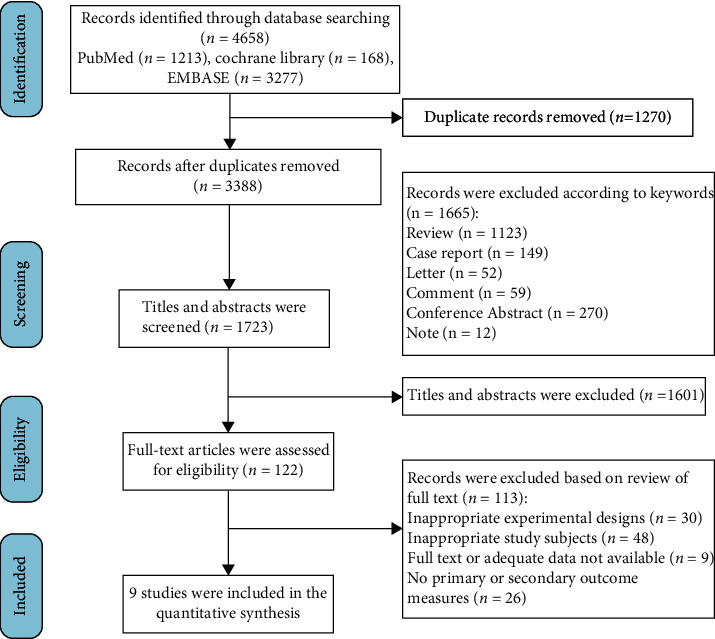
Flow diagram of the study selection process in the meta-analysis.

The process of literature search is outlined in Figure 1. Our initial search of three databases identified 4658 records: 1213 from PubMed, 168 from the Cochrane Library, and 3277 from Embase. Duplicate records were removed, and uncorrelated records were excluded according to keywords (review, case report, letter, comment, conference abstract, and note), after which 1601 were eliminated by title and abstract screening. The remaining 122 studies were accessed by reviewing the full text, after which 113 articles were excluded as they had inappropriate experimental design, inappropriate study subjects or outcome measures, or lacked full text or adequate data. Finally, nine studies that met the inclusion criteria were included in the quantitative synthesis.

The characteristics of these nine studies are presented in Table 1. The 9 studies involved 631 subjects. All of the nine studies included patients with ST-segment elevation myocardial infarction (STEMI; *n* = 4) [28, 29, 36, 37], ischemic heart diseases (IHD; *n* = 3) [27, 38, 39], acute coronary syndrome (ACS; *n* = 1) [40], and coronary heart disease (CHD; *n* = 1) [41]. The duration of melatonin administration ranged from 1 h and 1 min to 12 weeks. Melatonin was administered by intravenous or intracoronary injection or orally before surgery or after restoring the blood flow; the maximum single dose of melatonin was 50 mg and the minimum dose was 2 mg. The follow-up duration, referring to the maximum duration of outcome measurement, ranged from 45 min to 12 weeks. Four of the nine studies reported primary outcome measures (i.e., ventricular ejection fraction [LVEF], left ventricular end-diastolic volume [LVEDV], left ventricular end-systolic volume [LVESV], total LV mass, and infarct size), while the remaining five studies reported secondary outcome measures, namely, cardiovascular blood markers.

### 3.2. Study Quality

**Figure 2:**
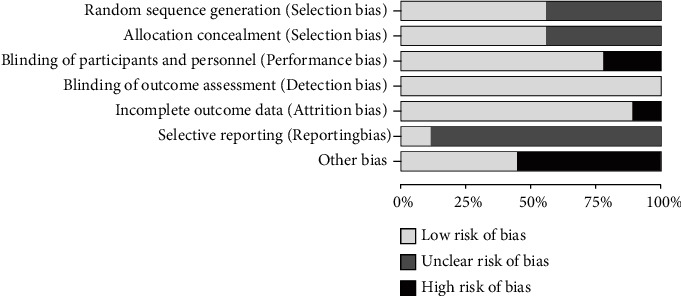
Risk-of-bias assessments of each of the included studies (domains from the Cochrane Handbook for Systematic Reviews of Interventions).

The risk of bias of the included studies is presented in Figure 2. All nine studies were randomized, and most of the studies (*n* = 5) described the specific methods of randomization. Double-blind (participants and personnel) was applied to almost all of the studies (*n* = 7), but only half described the methods of allocation concealment in detail. Because of the lack of double-blinding, two studies [37, 38] judged as “high” for performance bias. Although the concrete methods of blinding of outcome assessment were not mentioned, the objective measurement of cardiac function indicators and cardiovascular blood markers in these studies will not be greatly affected by the assessor factors. Therefore, the detection of bias was judged to be “low.” Owing to the lacking of intention to treat principles in the data analysis and balance for multiple variables between experimental and placebo groups after dropouts, only one study [37] was judged to have “high” for attrition bias. Specific experimental design data were unavailable in almost all of the included studies (*n* =8), which led to “unclear” risk of reporting bias. Studies (*n* = 5) were judged to have high risk of bias for other bias including those without a priori sample size analysis.

### 3.3. Main Efficacy of Meta-Analysis

#### 3.3.1. Primary Outcome Measures

**Figure 3:**
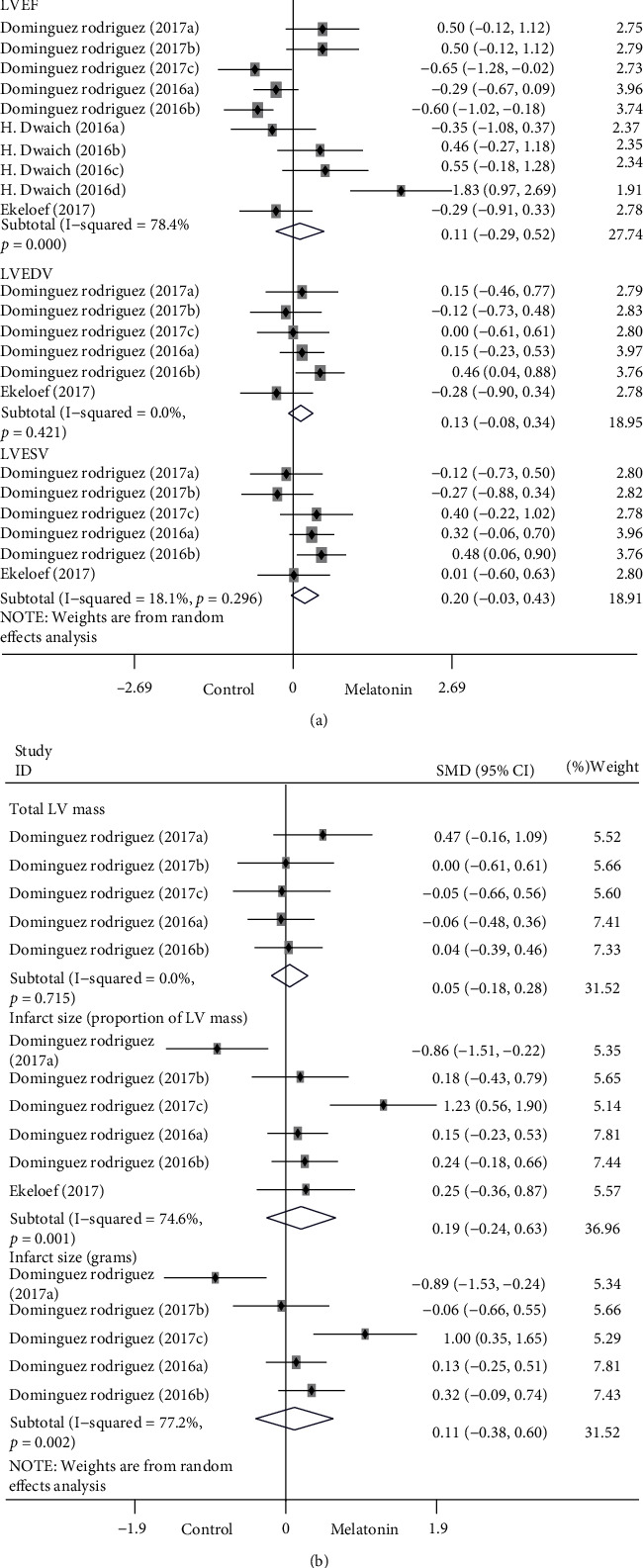
Forest plot of melatonin intervention on LV function, including LVEF, LVEDV, and LVESV (a), and myocardial infarct parameters, including total LV mass and infarct size (proportion of LV mass as well as grams) (b); CI: confidence interval, LV: left ventricular, LVEDV: left ventricular end-diastolic volume, LVEF: left ventricular ejection fraction, LVESV: left ventricular end-systolic volume, and *p*: heterogeneity *p*-value.

**Figure 4:**
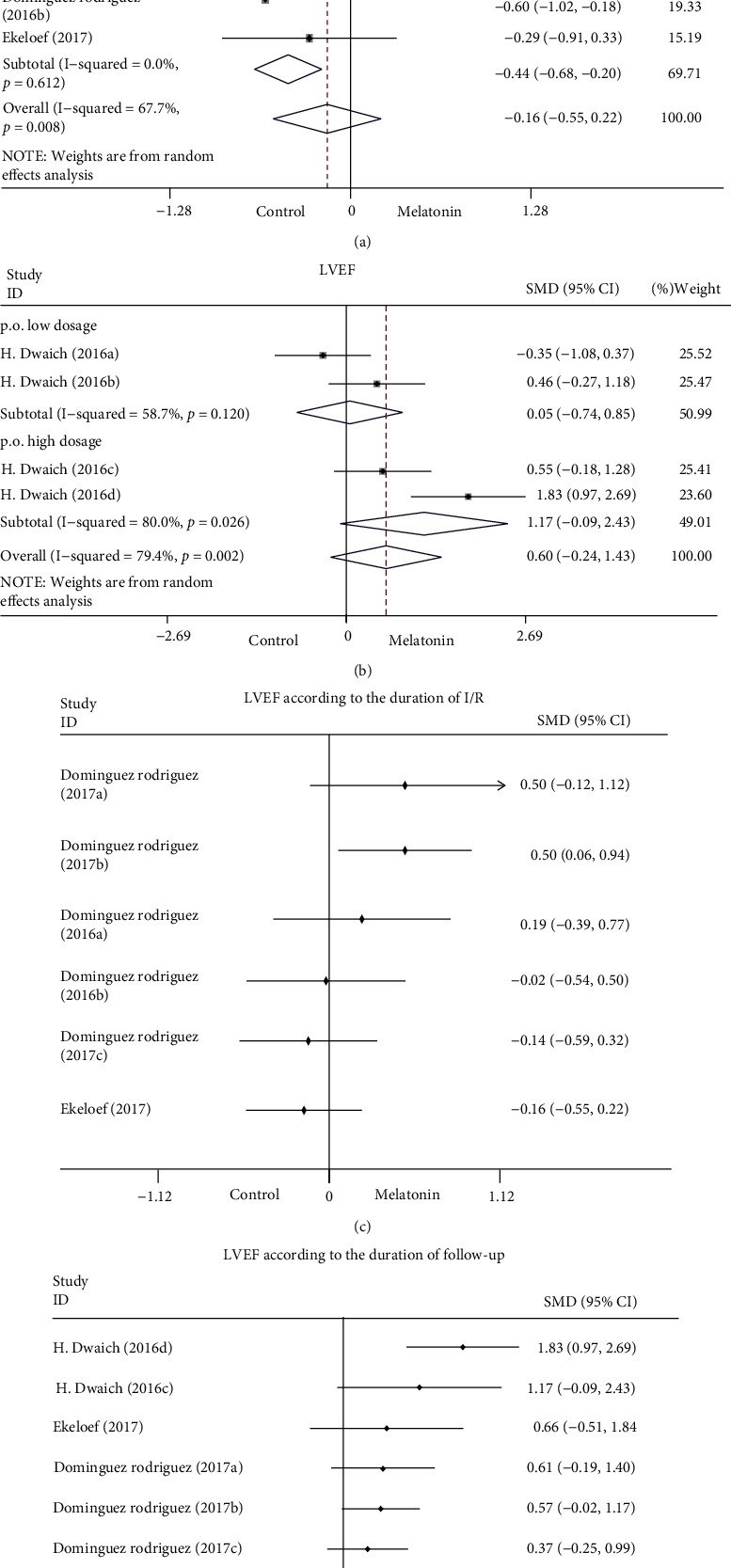
Forest plot of subgroup analysis according to the timing (a) and methods (b) of melatonin administration, and cumulative meta-analysis according to the duration of ischemia-reperfusion (c) and follow-up (d) on LVEF. CI: confidence interval, i.c.: intracoronary injection, i.v.: intravenous injection, I/R: ischemia-Reperfusion, p.o.: prally treated, and *p*: heterogeneity *p*-value.

*(1) Cardiac Function*. LV function, including LVEF, LVEDV, and LVESV, was reported in 4 studies involving 22 comparisons. The results suggested that melatonin intervention had no significant effects on LVEF, LVEDV, and LVESV compared to the control groups but showed a trend to enhance LVEF (SMD: 0.11; 95% CI: −0.29 to 0.52; *P* = 0.58; and *I*^2^ = 78.4%), LVEDV (SMD: 0.13; 95% CI: −0.08 to 0.34; *P* = 0.22; and *I*^2^ = 0%), and LVESV (SMD: 0.20; 95% CI: −0.03 to 0.43; *P* = 0.095; and *I*^2^ = 18.1%) in Figure 3(a). A high heterogeneity was revealed in the LVEF comparisons (*I*^2^ = 78.4%; *P* < 0.001). Sensitivity analysis by removing each study sequentially did not significantly influence the overall results. We conducted a subgroup analysis to investigate whether the methods and time of melatonin administration affected LV function and to establish potential heterogeneity. The results showed that LVEF was enhanced when melatonin was intravenous and intracoronary injection in the early stage of myocardial ischemia (<3.5 h; SMD: 0.50; 95% CI: 0.06 to 0.94; *P* = 0.03; *I*^2^ = 0.0%, Figure 4(a)). Inversely, LVEF was weakened (SMD: −0.44; 95% CI: −0.68 to −0.20; *P* < 0.001; *I*^2^ = 0%, Figure 4(a)) when melatonin was intravenous and intracoronary injection in the late stage of myocardial ischemia (≥3.5 h), and the heterogeneity decreased from 67.7% to 0%, which could be inferred from the methods and time of intervention. When melatonin was taken orally beforehand, there were no significant effects on LVEF, but there was a strong trend to promote LVEF (SMD: 0.60; 95% CI: −0.24 to 1.43; *P* = 0.16; and *I*^2^ = 79.4%, Figure 4(b)).

The results of cumulative meta-analysis showed that the effect of melatonin improving the left heart function diminished with the duration of ischemia-reperfusion (I/R) and may even worsen the left heart function. That is to say, the earlier that melatonin is administered, the more effective it is to boost the left heart function. In contrast, it may be harmful to the left heart function if it is administered later (Figure 4(c)). The results in Figure 4(d) indicated that the influence of melatonin enhancing LVEF declined with the duration of follow-up.

**Figure 5:**
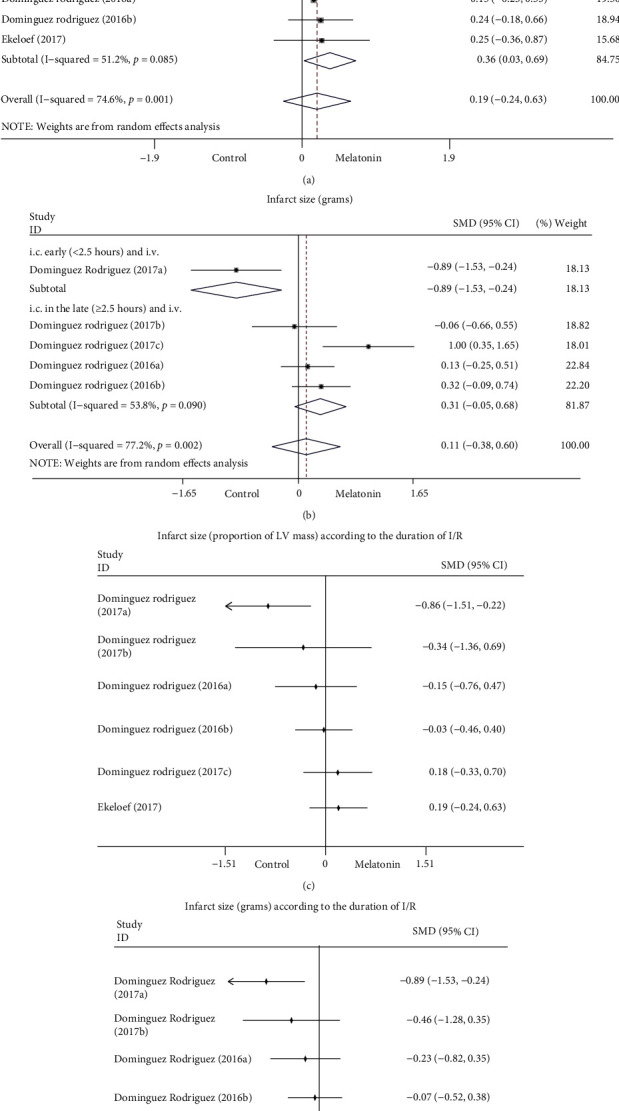
Forest plot of subgroup analysis according to the timing of melatonin administration on infarct size (proportion of the LV mass (a) as well as the grams (b)), and cumulative meta-analysis according to the duration of ischemia-reperfusion on infarct size (proportion of the LV mass (c) as well as the grams (d)). CI: confidence interval, i.c.: intracoronary injection, i.v.: intravenous injection, I/R: ischemia-reperfusion, p.o.: orally treated, and *p*: heterogeneity *p*-value.

*(2) Myocardial infarct parameters*. The assessment of myocardial infarct was expressed in included studies as total LV mass and infarct size. The infarct size was described as the proportion of LV mass and in grams. The final results indicated that melatonin treatment had no significant effects on total LV mass and infarct size (proportion of LV mass as well as grams) but suggested a trend toward increasing total LV mass (SMD: 0.05; 95% CI: −0.18 to 0.28; *P* = 0.68; and *I*^2^ = 0%), infarct size (grams; SMD: 0.11; 95% CI: −0.38 to 0.60; *P* = 0.66; and *I*^2^ = 77.2%) and infarct size (proportion of LV mass; SMD: 0.19; 95% CI: −0.24 to 0.63; *P* = 0.38; and *I*^2^ = 74.6%) in Figure 3(b). Sensitivity analysis by removing each study sequentially did not significantly influence the overall results. Subgroup analysis was performed to identify the sources of heterogeneity and whether the methods and time of melatonin administration influenced the infarct size. The forest plot showed similar results with LV function. The infarct size (proportion of LV mass) was enlarged when melatonin was injected into myocardial ischemia reperfusion at a late stage (≥2.5 h; SMD: 0.36; 95% CI: 0.03 to 0.69; *P* = 0.03; and *I*^2^ = 51.2%, Figure 5(a)). In contrast, melatonin injections at an early stage reduce the infarct size (<2.5 h; SMD: −0.86; 95% CI: −1.51 to −0.22; and *P* = 0.01, Figure 5(a)). Analogous trends were also observed in the subgroup analysis of infarct size (grams, Figure 5(b)), although the heterogeneity remained high. When we excluded the three sets of data from the Dominguez Rodriguez 2017 study, the heterogeneity was reduced to 0%. This indicated that the heterogeneity mainly originated from the study of Dominguez Rodriguez 2017 [29] and that the main difference between the three groups of data lies in the time of melatonin administration.

The results of cumulative meta-analysis demonstrated the effect of melatonin intervention on reducing the infarct size (proportion of LV mass, Figure 5(c); grams, Figure 5(d)) diminished with the duration of I/R and may even increase the infarct size when melatonin was administered later.

#### 3.3.2. Secondary Outcome Measures

**Figure 6:**
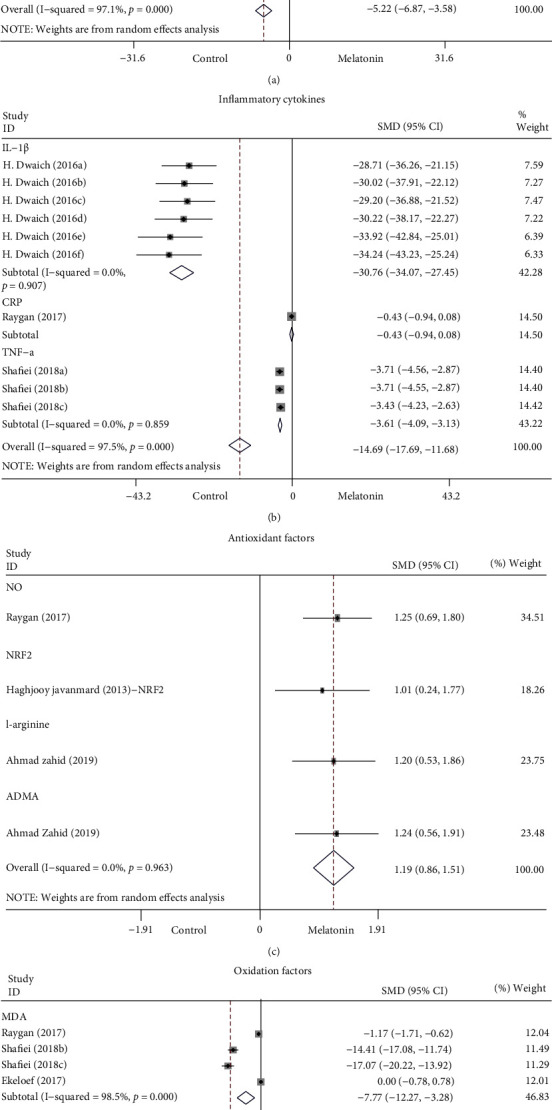
Forest plot of melatonin intervention on the level of cardiac injury markers (a), inflammatory cytokines (b), and antioxidant (c) and oxidation factors (d). ADMA: asymmetric dimethylarginine, CI: confidence interval, CKMB: creatine kinase myocardial band, CRP: C-reactive protein, hs-TnT: high-sensitive troponin T, IL-1*β*: interleukine-1*β*, iNOS: inducible nitric oxide synthase, MDA: malondialdehyde, NO: nitric oxide, NRF2: nuclear erythroid 2-related factor 2, *p*: heterogeneity *p*-value, and TNF-*α*: tumor necrosis factor-*α*.

The secondary outcome measures included cardiac injury markers, inflammatory cytokines, and oxidative stress indices. When supplied with melatonin, the forest plot showed a large effect on the level of cardiac injury markers (SMD: −5.22; 95% CI: −6.87 to −3.58; *P* < 0.001; and *I*^2^ = 97.1%, Figure 6(a)), inflammatory cytokines (SMD: −14.69; 95% CI: −17.69 to −11.68; *P* < 0.001; and *I*^2^ = 97.5%, Figure 6(b)), and oxidative stress indices. Therefore, we inferred that melatonin intervention prominently reduced the level of cardiac injury markers, inflammatory cytokines, and oxidation factors (SMD: 1.19; 95% CI: 0.86 to 1.51; *P* < 0.001; and *I*^2^ = 0%, Figure 6(c)), and markedly increased the level of antioxidant factors (SMD: −15.78; 95% CI: −19.96 to −11.61; *P* < 0.001; and *I*^2^ = 98.1%, Figure 6(d)). However, a high heterogeneity was exposed. Sensitivity analysis did not significantly affect the overall results, supporting the beneficial effects of melatonin.

## 4. Discussion

Based on an analysis of pooled data from the included RCTs, we found that melatonin intervention had no significant effects on cardiac function, including LVEF, LVEDV, and LVESV, and infarct size in patients with MIRI but showed a trend to protect cardiac function and reduce the infarct size compared to the control group. The overall results may be significantly influenced by the limited number of articles and the different regimens of melatonin administration. Furthermore, we found that route and timing regimen of melatonin administration remarkably influenced the results. When melatonin was administered by intravenous and intracoronary injection in early stage, melatonin showed the ability to protect cardiac function and reduce infarct size. Reversely, when melatonin was injected in the late stage, the results were the opposite and melatonin intervention may even deteriorate cardiac function. The overall results were showed in Graphical Abstract.

Two previous meta-analyses have demonstrated that melatonin therapy could improve MIRI [25, 26]. One meta-analysis demonstrated that melatonin therapy could significantly improve cardiac function and reduce infarct size in rodents after MIRI [25]. Another one revealed that melatonin administration had a favorable effect on LVEF in humans [26]. These conclusions do not quite consistent with ours. We included data of all comparisons including different route, timing, and dose regimen of melatonin administration, which interferes with the overall results but facilitate subgroup analysis to identify influencing factors. Theoretically, animal experiments contribute to the ability to explore biological mechanisms and offer some references for human studies. However, because of the large gap in the physiological structure and psychology between the different species, the extrapolation of results from animal to human studies is limited. Meanwhile, unique social attributes of humans and complex interference factors may be responsible for the inconsistent results between human and animal studies. Furthermore, differences in the timing of administration may also contribute to the inconsistency. In animal studies, melatonin was administered before myocardial I/R to achieve sufficient blood concentration in a particular site, whereas in humans with acute myocardial infarction it is difficult to predict and administer before myocardial I/R. However, because only four studies were involved in primary outcome measures, the insufficient sample size may have also led to differences in the results.

The subgroup analysis of LVEF and infarct size indicated that the route and timing regimen of melatonin administration remarkably affected its cardioprotective effect. When melatonin was administered by intravenous and intracoronary injection in the early stage of myocardial ischemia, the LVEF was improved (<3.5 h) and the infarct size was reduced (<2.5 h) compared to the control group. Meanwhile, the opposite results were observed when melatonin was administered by intravenous and intracoronary injection in the late stage of myocardial ischemia. A cumulative meta-analysis further demonstrated the relationship between the timing of administration and the improvement in LVEF. The earlier the administration of melatonin, the more significant the improvement, while later administration showed the reverse effect. Moreover, the effect decreased with follow-up time. MIRI may be associated with the process of LV remodeling during cardiac cell death, inflammation, fibrosis, electrophysiological remodeling, and vascular rarefaction, which leads to the deterioration of late cardiac function [42]. It is easy to infer that melatonin alleviates MIRI by reducing infarct size and thus improves LVEF. The results of one study included indicated that early treatment with melatonin reduced the infarct size in patients with STEMI after pPCI. Contrariwise, the infarct size was enlarged when melatonin was administered at the late stage of myocardial ischemia [29]. Similarly, in the closed-chest porcine model of MIRI, the author concluded that melatonin treatment did not reduce MIRI, which may be related to the timing of melatonin administration [43]. A previous study has demonstrated that the mechanisms of myocardial cell death induced by reperfusion differ based on the duration of ischemia [44]. Therefore, we hypothesized that early melatonin administration was more mechanically-targeted and showed a greater protective effect. Indeed, in the early stage of myocardial ischemia, the oxidative stress is latent, and melatonin can effectively reduce infarct size. In contrast, in the late stage, the oxidative stress is irreversible, and melatonin cannot effectively reduce infarct size, and, as a fibrotic agent, may even increase the infarct area [45]. Therefore, we tend to recommend that melatonin is injected in the early stage of myocardial ischemia. Owing to the limited number of studies, the exact definition of the early and late period is ambiguous and inexact. Based on the available evidence, we have artificially defined early and late. The time limit of the early stage was different for LVEF and infarct size. Therefore, it is important to find the exact and appropriate dividing line. We expect further relevant studies to explore the underlying laws and mechanisms. Furthermore, the results were slightly different when infarct size was measured in grams and proportion of LV mass. Measuring infarct size in grams alone may be affected by differences in heart mass among individuals. Therefore, we tend to measure infarct size in proportion of LV mass.

The finding that melatonin intervention tended to enhance LV end-diastolic and end-systolic volume indicated that melatonin has a deleterious effect on LV remodeling [28]. Early ventricular remodeling is considered an adaptive response to protect cardiac function. It has been shown that melatonin enhances the accumulation of myocardial extracellular matrix (collagen and glycosaminoglycan) in infarcted myocardium [45, 46]. One study suggested that this phenomenon may be related to the dose of melatonin administration and suggested that too high serum concentration of melatonin may result in a loss of cardioprotection [28]. Three studies that selected intravenous or intracoronary injection all used doses of melatonin >1,000 times higher than that present in nocturnal blood levels. Therefore, more-rigorous studies are needed to determine the optimal dose of melatonin administration.

Differences were also observed in the methods of melatonin administration in this analysis. The results of one trial involving elective patients undergoing CABG without active acute response chose oral therapy with melatonin before surgery indicated that melatonin treatment improved LVEF and eased MIRI in a dose-dependent manner [27]. The results of the subgroup analysis also confirmed this trend. As for acute patients with MI, it is difficult to predict and adopt methods of administration in advance. Therefore, the remaining three studies involved patients with STEMI who opted for intravenous or intracoronary injection of melatonin [28, 29, 36]. For selective patients, oral administration may be an appropriate option; however, owing to only one study involving selective patients using oral therapy, it is difficult to determine whether oral or intravenous and intracoronary administration is better. Thus, we expect more relevant studies to identify the optimal method, timing, and dosage of melatonin treatment in the future.

Many studies have identified the cardioprotective function of melatonin to alleviate MIRI and explored the underlying mechanisms, which mainly comprise its powerful free radical scavenging ability, regulation of antioxidant systems, and a series of signaling pathways [47–49]. Among them, the antioxidant effect of melatonin has always been the most attractive. Melatonin and its major metabolites have their own direct free radical scavenging abilities via hydrogen atom and single electron transport [50, 51]. Meanwhile, melatonin has been shown to increase the expression of antioxidant enzyme genes and inhibits pro-oxidative enzymes [52]. The powerful antioxidant capacity of melatonin has been shown to contribute to reduce the infarct size and improve myocardial systolic function during myocardial I/R [22]. Moreover, accumulated evidence suggested that melatonin eased MIRI through many of its other properties, including anti-inflammation, modulating autophagy, mitochondrial function, and antiapoptosis effects [53, 54]. In the current meta-analysis, cardiac injury markers, inflammatory cytokines, and oxidation factors were significantly eased after melatonin intervention, which further proved its cardioprotective function. Cardiac troponin I, high-sensitive Troponin T (hs-TnT), and the creatine kinase-MB (CK-MB) are sensitive biomarkers for detecting myocardial cell injury [55, 56]. Therefore, from the perspective of biological markers, it can be inferred that melatonin can reduce myocardial cell injury after I/R. Melatonin treatment also significantly improved inflammatory factors and biomarker oxidative stress, which further demonstrated its anti-inflammatory and antioxidant power. Despite the high heterogeneity, sensitivity analysis by removing each study sequentially did not significantly influence the overall results. The heterogeneity may derive from different measurement timings and biological indicators. As part of MIRI mechanisms, inflammation and oxidative stress were ameliorated by melatonin administration, which reduced myocardial damage [57, 58].

Two studies have reported on the safety of melatonin. One study used a melatonin blood level that was approximately 12,000 times higher than the highest nocturnal blood level reported no significant differences in the incidence of adverse outcomes (reinfarction, occurrence of angina, congestive heart failure, and death) during the 12-month follow-up period [28]. Intravenous injection of melatonin has been demonstrated to be nontoxic to humans in previous trials [59]. Another study also indicated that melatonin treatment had no adverse effects on patients [36]. Therefore, we believe melatonin to be a safe and well-tolerated treatment.

Several potential limitations should be considered. First, due to the results of our meta-analysis were based on study-level data rather than individual participant level data which impeded further subgroup analysis. It is difficult to analyze the effect of sex, age, comorbidities, and other factors on the cardioprotective effects of melatonin. Second, the amount of human research on primary outcomes was limited, which inevitably affected the main results and limited subsequent subgroup analyses to explore further underlying influencing factors. Third, we used digital software to acquire data, which may lead to a certain degree of data error. Fourth, the time of myocardial ischemia and the route, dosage, and timing regimen of melatonin administration varied between studies, which interfered to some extent with the main outcomes and led to less convincing results. This makes it difficult to deduce the optimal regimen for melatonin administration. Fifth, objective quantitative statistics on cardiovascular adverse events are limited. Therefore, larger and more well-designed RCTs are needed to determine more precise results and optimal schedules of melatonin administration.

## 5. Conclusion

Our meta-analysis showed that melatonin intervention had no significant effects on cardiac function and infarct size in patients with MIRI; however, the result was significantly influenced by the limited number of included studies. Subgroup analyses found that the cardioprotective function of melatonin for MIRI was influenced by the route and timing regimen of melatonin administration. We tend to support that melatonin is injected early in acute patients. Moreover, melatonin improves the level of cardiac injury markers and has anti-inflammatory and antioxidant effects. Additional more well-designed RCTs are needed to further identify the optimal timing regimen of melatonin administration and the detailed mechanisms of cardioprotective effects of melatonin.

## Figures and Tables

**Table 1:** Characteristics of the included studies.

Author(s)	Country	Sample size	Age (mean; years)	Diagnosis	Dose and time of melatonin administration	IR duration (min)	Follow-up (*d*)	Primary outcome measure	Secondary outcome measure
Alberto Dominguez-Rodriguez, 2016a	Canarias	55/53	57.3 ± 10 58.4 ± 9.4	STEMI	12 mg i.v. before pPCI and 2 mg i.c. after restoring the blood flow	200	6 ± 2	LVEF, LVSDV, LVEDV, and total LV mass	—
Alberto Dominguez-Rodriguez, 2016b	Canarias	44/47	57.3 ± 10 58.4 ± 9.4	STEMI	As above	200	130 ± 10	As above	—
Alberto Dominguez-Rodriguez, 2017a	Spain	19/22	54 ± 10 57 ± 9	STEMI	As above	136 ± 23	7	As above	—
Alberto Dominguez-Rodriguez, 2017b	Spain	25/18	58 ± 10 56 ± 10	STEMI	As above	196 ± 19	7	As above	—
Alberto Dominguez-Rodriguez, 2017c	Spain	21/20	60 ± 11 59 ± 8	STEMI	As above	294 ± 41	7	As above	—
Ebrahim Shafiei, 2018	Iran	30/30	62.0 ± 8.8 61.6 ± 7.7	IHD	5 mg p.o. from 24 h before the CABG, three times	—	After recovery at the ICU	—	Troponin I, MDA, and TNF-*α*
Fariba Raygan, 2017	Iran	30/30	67.7 ± 11.4 65.3 ± 10.1	CHD	10 mg/day 12 weeks	—	12 weeks	—	MDA, hs-CRP, and NO
Jawad Ahmad Zahid, 2019	Denmark	15/16	65.3 ± 7.4 63.9 ± 9.1	ACS	25 mg p.o. for 12 weeks following ACS	—	84 days	—	l-arginine and ADMA
Karar H. Dwaich, 2016a	Iraq	15/15	52.3 ± 6 52.5 ± 3.6	IHD	10 mg/day p.o. for 5 days before CABG	—	1 day	LVEF	cTn-I, IL-1*β*, and iNOS
Karar H. Dwaich, 2016b	Iraq	15/15	53.9 ± 6.1 52.5 ± 3.6	IHD	20 mg/day p.o. for 5 days before CABG	—	1 day	LVEF	cTn-I, IL-1*β*, and iNOS
Padideh Ghaeli, 2015	Iran	20/20	58.64 ± 12.91 58.13 ± 11.87	STEMI	3 mg p.o.		6 h after pPCI	—	Hs-TnT and CKMB
Sarah Ekeloef, 2017	Denmark	22/19	61.7 ± 2.65 64.0 ± 2.40	STEMI	1 mg i.c. after restoring the blood flow and 49 mg i.v. after pPCI	<360	4 ± 1 day	LVEF, LVSDV, LVEDV, infarct size	Hs-TnT, CKMB, AOPP, MPO, and MDA
Shaghayegh Haghjooy Javanmard, 2013	Iran	15/15	58.1 ± 9.8 60.1 ± 9.2	IHD	10 mg p.o. before sleeping for 1 month before CABG	—	45 min after the operation	—	NRF2

ACS = acute coronary syndrome. ADMA = asymmetric dimethylarginine. AOPP = advanced oxidation protein products. CABG = coronary artery bypass grafting. CHD = coronary heart disease. CKMB = creatinine kinase myocardial band. cTn-I = cardiac troponin I. hs-CRP = high sensitivity C-reactive protein. hs-TnT = high-sensitive troponin T. IR = ischemia reperfusion injury. i.c. = intracoronary injection. i.v. = intravenous injection. ICU = intensive care unit. IHD = ischemic heart disease. IL-1*β* = interleukine-1*β*. iNOS = inducible nitric oxide synthase. LV = left ventricular. LVEF = left ventricular ejection fraction. LVESV = left ventricular end-systolic volume. LVEDV = left ventricular end-diastolic volume. MDA = malondialdehyde. MPO = myeloperoxidase. NRF2 = nuclear erythroid 2-related factor 2. NO = nitric oxide. p.o. = orally treated. pPCI = primary percutaneous coronary intervention. STEMI = ST-segment elevation myocardial infarction.
